# Therapeutic Effect of Long-Term Epidural Block in Rats with Pregnancy Induced Hypertension

**DOI:** 10.1155/2018/1639623

**Published:** 2018-02-07

**Authors:** Nianjiao Han, Yang Li, Youjing Dong

**Affiliations:** Department of Anesthesia, Shengjing Hospital of China Medical University, No. 36, Sanhao Street, Herping District, Shenyang 110004, China

## Abstract

**Background:**

Pregnancy induced hypertension (PIH) causes a variety of systemic disorders that negatively affect the maternal placenta and fetal growth. Epidural sympathetic block elicits symptoms of decreased blood pressure. This study was designed to determine the therapeutic effect of long-term epidural block in rats with PIH.

**Methods:**

Forty healthy pregnant Sprague Dawley rats were randomized into four groups with each group consisting of 10 rats. On gestation day (GD) 14, rats in control group underwent a sham procedure; rats in RUPP group were operated on to obtain reduced uterine perfusion pressure (RUPP); rats in RUPP plus normal saline (NS) group were also subjected to the RUPP procedure and underwent epidural block with 25 *μ*l normal saline twice daily until delivery; rats in RUPP plus epidural block (EB) group were treated as those in RUPP plus NS group except that an epidural block with 25 *μ*l of 0.125% bupivacaine was administered two times per day until delivery. On GD 20, blood pressure was measured in all groups before delivery, and blood samples were collected in order to quantify the serum concentrations of vascular endothelial growth factor (VEGF) and soluble fms-like tyrosine kinase 1 (sFlt-1).

**Results:**

The mean arterial pressure (MAP) of rats in RUPP group (147.6 ± 6.0 mmHg) was markedly increased when compared with control group (80.8 ± 4.6 mmHg) (*p* < 0.05). The MAP of rats in RUPP plus EB group (114.4 ± 7.2 mmHg) was clearly decreased in contrast with RUPP group but was still higher than in control group (*p* < 0.05). The variation of fetal weight in all groups followed a similar trend to that of MAP. However, there were no significant differences between control group and RUPP plus EB group with respect to placental weight (*p* = 0.186). Variation in MAP was positively correlated with the expression of sFlt-1 in each group but was negatively correlated with VEGF.

**Conclusion:**

This study demonstrates that long-term epidural block decreases blood pressure in PIH rats and improves the serum concentrations of VEGF and sFlt-1. Taken together, long-term epidural block may have a potential role in PIH treatment.

## 1. Introduction

Pregnancy induced hypertension (PIH) is one of the most severe syndromes during pregnancy and is a major contributor to maternal and perinatal mortality and morbidity [1, 2]. The pathophysiology and pathogenetic mechanisms of PIH remain unclear. It has been hypothesized [3] that placental ischemia is involved in the pathogenesis of PIH and in hypertensive pregnancies in which the trophoblast invasion is abnormal and the physiological conversion of spiral arteries is limited to the decidua. This causes maternal endothelial dysfunction and reduced uteroplacental perfusion, which can lead to a complex disorder involving hypertension, proteinuria, and intrauterine growth restriction [4].

Placental ischemia may result in the release of placental materials, such as vascular endothelial growth factor (VEGF), into circulation. The expression of VEGF is observed in many tissues but is particularly pronounced at the vascular bud site on the surface of placental syncytiotrophoblast cells during early pregnancy [5, 6]. Overexpression of VEGF leads to vascular endothelial dysfunction and it was observed that the concentration of serum VEGF is increased in preeclampsia compared to normal pregnancy [7]. As a high affinity receptor of VEGF, soluble fms-like tyrosine kinase 1 (sFlt-1) is also present in extravillous trophoblasts. A study has indicated [8] that the concentration of sFlt-1 in the maternal placenta of preeclampsia is greater than normal and that it is positively correlated with the severity of preeclampsia.

Epidural block is an anesthesia method that is widely used in the clinic. The specific approach in epidural anesthesia is to inject drugs into the epidural space bordering on the spinal dura mater to achieve nerve blocking. Long-term epidural analgesia is a critical tool in postoperative pain management [9].

To date, there is no effective treatment for PIH except early delivery of the neonate. Epidural block can reduce blood pressure and heart rate by blocking thoracic sympathetic, thereby alleviating myocardial oxygen demand and, furthermore, regulating the metabolic function of cardiac tissue [10], which may be helpful for patients with PIH. We hypothesized that long-term epidural block may be a potential method to reduce blood pressure in pregnant women with PIH.

The purpose of this study was to determine the therapeutic effects of long-term epidural block on blood pressure and to evaluate the serum concentrations of VEGF and sFlt-1 in rats with PIH in order to identify a new strategy for clinical treatment.

## 2. Materials and Methods

### 2.1. Ethics Statement

This project was approved by the Animal Care and Use Committee of China Medical University Institution (IACUC number 2015214) and it complied with all guidelines for animal welfare.

### 2.2. Animals and Groups

The study involved forty healthy Sprague Dawley rats (Benxi Changsheng Biotechnology Co., Ltd., China). Day of appearance of the sperm plug was marked as gestational day (GD) 0. The rats were housed at 20–25°C on a 12 : 12 h light-dark cycle. They were fed with food and water ad libitum.

The 40 pregnant rats were randomly divided into four groups: control group, reduced uterine perfusion pressure (RUPP) group, RUPP plus NS group, and RUPP plus EB group. On GD 14, the rats in control group underwent a sham procedure; rats in RUPP group were subjected to a mechanical procedure to obtain reduced uterine perfusion pressure; rats in RUPP plus NS group were subjected to a procedure to obtain RUPP and underwent epidural block with 25 *μ*l normal saline twice daily until delivery; rats in RUPP plus EB group were treated as in RUPP plus NS group except that epidural block was achieved with 25 *μ*l of 0.125% bupivacaine twice daily until delivery.

RUPP is the method of choice for establishing PIH rat models and was applied in this study. On GD 14, all rats were anesthetized with 10% chloral hydrate before undergoing surgical procedures. To expose the lower abdominal aorta and ovarian aorta adequately, an abdominal incision of 2.5–3 cm was made after strict disinfection in all groups. Then, the abdominal aorta iliac artery bifurcation and the first branches of both the left and right ovarian arteries were identified, and these arteries were narrowed down to 0.33 mm to limit their supply to the uterus and to achieve placental ischemia in all rats except for those in control group. The rats in RUPP plus NS group and RUPP plus EB group also required an epidural catheter for epidural block before regaining consciousness. After the completion of the operations, rats in each group were housed separately until delivery.

### 2.3. Blood Pressure Measurements

On GD 19, after anesthesia with 10% chloral hydrate, indwelling catheters were implanted into the carotid arteries of rats in all groups and fixed in the neck. Heparin mixed with saline solution was injected into the catheters in order to avoid blood clotting. A pressure transducer that was connected to catheters monitored blood pressure after zero adjustment and recorded continuously for 10 min after stable induction of anesthesia.

### 2.4. Blood Sampling and Analysis of VEGF and sFlt-1

On GD 19, caesarean section was performed in all rats under anesthesia. The weights of each fetus and placenta were recorded. Blood samples were collected from each rat and were centrifuged in 3000*g* lasting 5 minutes to get serum that was then stored at −70°C until analysis for VEGF and sFlt-1. The serum concentrations of VEGF and sFlt-1 were detected by enzyme-linked immunosorbent assay according to the manufacturer's instructions.

### 2.5. Statistical Analysis

The results are showed as mean ± SD. Statistical analysis was performed by IBM SPSS Statistics for MAC, version 24 (IBM Corp., Armonk, NY, USA). An independent sample *t*-test was used for comparison between two groups and one-way ANOVA test with post hoc Tukey HSD test was applied for analysis among four groups. *p* < 0.05 was considered statistically significant.

## 3. Results

**Figure 1:**
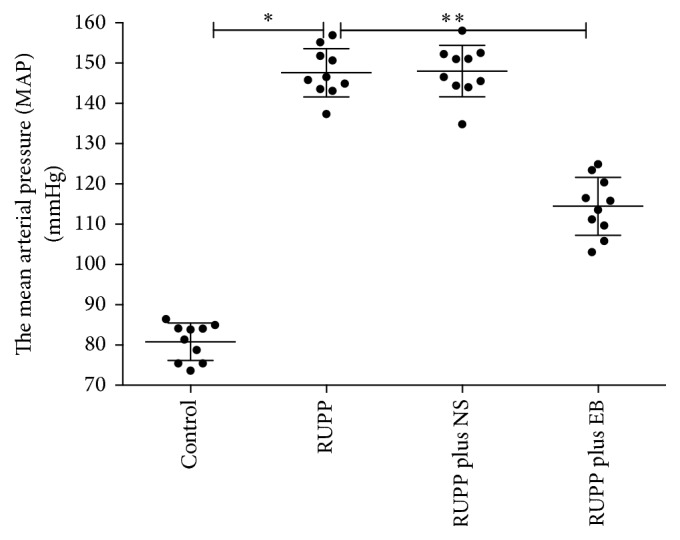
*Scattergram illustrating* the mean arterial pressure (MAP) of each rat in all groups. The data are expressed as mean ± SD. ^*∗*^*p* < 0.05, the MAP of rats in control group (80.8 ± 4.6 mmHg) versus the MAP of rats in RUPP group (147.6 ± 6.0 mmHg); ^*∗∗*^*p* < 0.05, the MAP of rats in RUPP group (147.6 ± 6.0 mmHg) versus the MAP of rats in RUPP plus EB group (114.4 ± 7.2 mmHg).

As presented in Figure 1, the mean arterial pressure (MAP) of rats in control group was much lower than in the other three groups (*p* < 0.05). There were no significant differences between RUPP group (147.6 ± 6.0 mmHg) and RUPP plus NS group (148.0 ± 6.4 mmHg). However, the MAP of rats in RUPP plus EB group was lower than in RUPP group and RUPP plus NS group (*p* < 0.05). The MAP of rats in RUPP plus EB group was slightly elevated compared to the MAP of rats in control group (*p* < 0.05).

**Figure 2:**
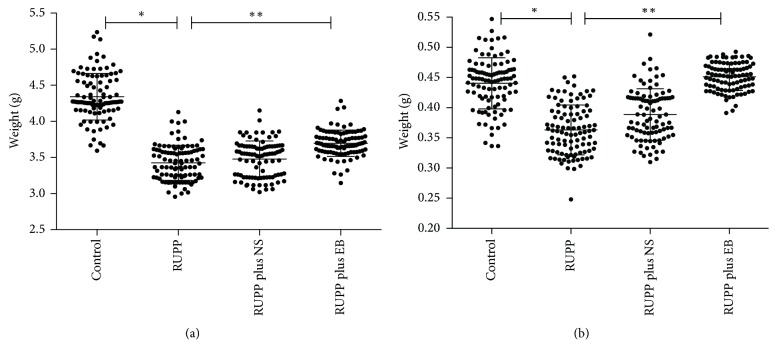
*Scatterplot of* the fetal parameters of rats in all groups. The data are expressed as mean ± SD. The fetal weight of rats in all groups was present in (a). ^*∗*^*p* < 0.05, the fetal weight of rats in control group (4.34 ± 0.19 g) versus the fetal weight of rats in RUPP group (3.42 ± 0.10 g); ^*∗∗*^*p* < 0.05, the fetal weight of rats in RUPP group (3.42 ± 0.10 g) versus the fetal weight of rats in RUPP plus EB group (3.69 ± 0.09 g). The placental weight of rats in all groups was present in (b). ^*∗*^*p* < 0.05, the placental weight of rats in control group (0.44 ± 0.02 g) versus the placental weight of rats in RUPP group (0.36 ± 0.02 g); ^*∗∗*^*p* < 0.05, the placental weight of rats in RUPP group (0.36 ± 0.02 g) versus the fetal weight of rats in RUPP plus EB group (0.45 ± 0.01 g).

Figure 2 shows fetal parameters including the average weight of each fetus and corresponding placental weight in all groups. It was evident that, similarly to the MAP, the fetal weight in control group rats was higher than in RUPP group and RUPP plus NS group (*p* < 0.05). The fetal weight in RUPP plus EB group was modestly increased compared with RUPP group (*p* < 0.05) but was not as high as in control group (*p* < 0.05). In contrast to fetal weight, the placental weight in RUPP group rats was slightly lower than in control group rats (*p* < 0.05) and there was no significant difference between control group (0.44 ± 0.02 g) and RUPP plus EB group (0.45 ± 0.01 g).

**Figure 3:**
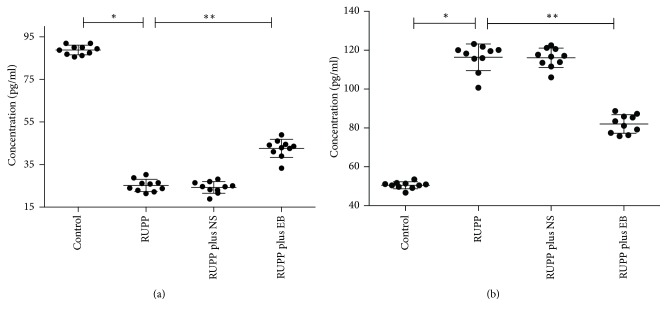
The serum parameters of rats in all groups. The data are expressed as mean ± SD. The concentration of VEGF in all groups was present in (a). ^*∗*^*p* < 0.05, the VEGF of rats in control group (88.2  ±  2.3 pg/ml) versus the VEGF of rats in RUPP group (25.2  ±  2.9 pg/ml); ^*∗∗*^*p* < 0.05, the VEGF of rats in RUPP group (25.2  ±  2.9 pg/ml) versus the VEGF of rats in RUPP plus EB group (42.6 ± 4.2 pg/ml). The concentration of sFlt-1 in all groups was present in (b). ^*∗*^*p* < 0.05, the sFlt-1 of rats in control group (50.5 ± 1.8 pg/ml) versus the sFlt-1 of rats in RUPP group (116.4  ±  6.9 pg/ml); ^*∗∗*^*p* < 0.05, the sFlt-1 of rats in RUPP group (116.4  ±  6.9 pg/ml) versus the sFlt-1 of rats in RUPP plus EB group (82.1  ±  4.7 pg/ml).

All the serum parameters are presented in Figure 3. The expression of VEGF in rats in control group was significantly higher than in the other three groups (*p* < 0.05). The concentration of VEGF in RUPP plus EB group rats was also elevated compared to rats in RUPP group (*p* < 0.05). However, the levels of sFlt-1 exhibited an opposing trend to that of VEGF and were markedly lower in control group rats compared with the other three groups (*p* < 0.05). Further, the concentration of sFlt-1 in RUPP plus EB group rats was significantly less than in RUPP group (*p* < 0.05).

## 4. Discussion

The purpose of the experiments described in this study was to explore the therapeutic effects of long-term epidural block on the symptoms of PIH, that is, changes in MAP and the concentration of VEGF and sFlt-1. The results of the study show that the MAP of rats subjected to the RUPP method was much higher than the rats of the control group and that, compared to the MAP of rats subjected to the RUPP method, the MAP of rats with long-term epidural block was significantly reduced. Meanwhile, the serum concentration of sFlt-1 in rats subjected to the RUPP method was markedly elevated compared to both the control group and rats given long-term epidural block. These results demonstrate that the RUPP method was very successful in establishing a PIH rat model and show that long-term epidural block may alleviate the symptoms PIH to a certain extent.

The RUPP method was used to generate the rat model of PIH in our study. By reducing the perfusion of the uterine placenta, the RUPP method ensures that maternal circulation cannot fully flood the uterine spiral artery. This series of processes leads to placental ischemia and is due to elevated blood pressure as well as other effects [11, 12]. This RUPP model has been performed in a variety of different animals including dogs, rabbits, sheep, and rats. Of all the animal models, the RUPP pregnant rat model is the most practical method and has been most comprehensively studied [13]. Intapad et al. [14] show that reduced uterine perfusion pressure in the pregnant mouse induces hypertension while probing the mechanistic pathogenesis of preeclampsia. Their study indicates that placental ischemia is closely related to an elevation of blood pressure and fetal growth restriction. These evidences demonstrate that RUPP models in rats can be used as an alternative method to study preeclampsia. Schenone et al. [15] also carried out similar experiments in order to rigorously determine the practical use of the RUPP model in rats. They concluded that a selective reduced uterine perfusion pressure model in rats is very similar to PIH in humans and represents the best method for studying preeclampsia. In our study, as in previous research, the MAP of rats that had undergone the RUPP operation was far higher than rats in the control group and the magnitude of this increase was similar to other studies [14] on PIH or preeclampsia in which a rat RUPP model was used. Similar findings were obtained for serum factors like VEGF. Therefore, it can be concluded that the rat RUPP model described here successfully mimicked PIH.

In this study, fetal weight of rats undergoing the RUPP operation was markedly lower compared to the control group. This value was slightly greater in rats with long-term epidural block but was still lower than normal. These results corroborate [16, 17] other preeclampsia rat models, which reported a correlation between fetal number and weight. This does not simply indicate the success of the PIH rat model; the marginal increase of fetal weight also proves that long-term epidural block in PIH rats can alleviate the symptoms of fetal growth restriction to some extent. Nevertheless, the data on placental weight were in marked contrast to these results. Although the placental weight of rats after the RUPP operation was significantly lower than in the other three groups, the data demonstrated that there was no difference between the placental weight of rats with long-term epidural block and the control group. The results suffice to show that long-term epidural block can relieve placental ischemia caused by constriction of the uterus and ovarian arteries. We assume that the additional blood flow allowed the placenta to achieve a normal weight resulting in improved fetal weight, thereby alleviating fetal growth restriction.

VEGF is a potent angiogenesis factor and plays an important part in reducing vascular tone and blood pressure [18]. VEGF receptors are found in the endothelium where they act to increase cell proliferation. VEGF is also involved in the formation, control, and recovery of the vascular system [19]. Moreover, VEGF is the most abundant vascular growth factor in the maternal placenta during pregnancy, also modulates the immune system, and has been found to be involved in the pathology of PIH [20, 21]. Research by Wang et al. [22] found a close relationship between VEGF and PIH. Their findings suggest that the expression of VEGF in PIH is markedly lower compared to normal pregnancies and revealed a negative correlation between the severity of the disease and the expression of VEGF; that is, the more serious the condition, the lower the concentration of VEGF. Gilbert et al. [23] also found that the level of free VEGF in rats that had undergone the RUPP procedure was markedly reduced compared to normal, which is in agreement with the results of our experiments. However, while the concentration of VEGF in RUPP rats after long-term epidural block showed a clear recovery, the levels were still lower than in the control group.

In pregnancy, placenta-derived sFlt-1 (soluble fms-like tyrosine kinase 1) acts as a VEGF-trap reducing free VEGF availability for binding to its receptors. A number of studies [24, 25] have found sFlt-1 to be overexpressed in the endothelial cells of preeclamptic pregnancies in contrast to normal. This protein is related to the pathogenesis of preeclampsia and is a receptor of VEGF; sFlt-1 begins to rise in the early stages of preeclampsia and is positively correlated with disease severity. Other studies [26, 27] have found that when sFlt-1 was chronically injected into healthy pregnant rats it led to an elevation of MAP and a reduction of fetal weight, which supports the theory that sFlt-1 dysregulation is involved in the pathogenesis of preeclampsia. In this study, we observed that MAP decreased together with the concentration of sFlt-1 in response to long-term epidural block, suggesting that high sFlt-1 levels could be one of the causes of the pathogenesis of preeclampsia and that long-term epidural block has a certain therapeutic effect in rats with PIH. Spradley et al. [28], based on their series of experiments, concluded that there is a marked reduction of VEGF in RUPP rats and that the concentration of sFlt-1 is elevated in rats with placental ischemia-induced hypertension. Studies performed by other researchers [29] have yielded similar results.

Recent studies [30] have described a state of sympathetic overactivity in normotensive pregnant women and showed that such a condition is even more pronounced in women with PIH or preeclampsia. Based on data generated by intramural microelectrodes in the blood vessels of skeletal muscle, Fischer et al. [31] concluded that the excitability of the sympathetic nerve in preeclampsia women is more than threefold higher than normal pregnant women. The state of sympathetic overactivity in pregnant women returns to normal after delivery, but there is no change in sympathetic nerve activity in hypertensive nonpregnant women. The trace local anesthetic used in epidural block can selectively block the sympathetic nerve. Low concentrations of bupivacaine can elicit an extended sympathetic block, which is caused by thoracic epidural analgesia [32]. Inhibition of the sympathetic nerve can lead to vessel dilation, increased blood flow, and inhibition of smooth muscle after the spread of local anesthetics in the epidural space. Therefore, long-term epidural block not only can depress sympathetic overactivity but can also alleviate the clinical symptoms of PIH.

To the best of our knowledge, this is the first study to perform long-term epidural block on rats with RUPP and to report that this procedure can alleviate the symptoms placental ischemia-induced hypertension in rats. The limitations of this study are mainly linked to two aspects; firstly proteinuria was not detected in this study. The lack of urine protein may confound our results. However, there are recent indications [33] that proteinuria is no longer a prerequisite for a diagnosis of preeclampsia and that it can be replaced by alternative symptoms. A further limitation is that the relationship between sympathetic nerve activity and VEGF or sFlt-1 in RUPP rats with long-term epidural block is limited. More evidence is needed to determine whether the inhibition of sympathetic nervous excitability caused by long-term epidural block has any influence on serum VEGF and sFlt-1. Notably, as a side effect of epidural block, total spinal block and local anesthetic toxicity caused by mistakenly injecting local anesthetic into the cerebrospinal fluid or blood vessel occur infrequently in a clinical setting. This side effect did not occur in our study.

In summary, this study has demonstrated that, compared to rats with placental ischemia-induced hypertension, the MAP, VEGF, sFlt-1, fetal weight, and placental weight were slightly improved in RUPP rats with long-term epidural block. These results suggest that long-term epidural block may in part relieve the symptoms of PIH. However, further studies are needed to conclusively demonstrate whether long-term epidural block could be used in the clinical treatment of PIH.
